# A High-Sensitivity Relative Humidity and Temperature Fiber Optic Sensor Based on a Chitosan-Coated Mach-Zehnder Interferometer

**DOI:** 10.3390/mi17060652

**Published:** 2026-05-25

**Authors:** Jiangyu Qu, Yu Guo, Haidong Shao, Ruihong Xiong, Jiayi Xuan, Ruoning Wang, Cuiting Sun

**Affiliations:** College of Science, Northeast Forestry University, Harbin 150001, China

**Keywords:** Mach-Zehnder interferometer, relative humidity, temperature measurement, chitosan coating

## Abstract

In this work, we propose a bamboo-shaped Mach-Zehnder interferometer coated with chitosan for relative humidity (RH) and temperature measurement. The sensor is fabricated by fusing no-core fiber and multimode fiber segments through arc discharge, followed by tapering with a hydrogen–oxygen flame to form a unique bamboo-shaped configuration. To functionalize the structure for humidity sensing, chitosan is coated onto the fiber surface. The refractive index of chitosan varies with water molecule adsorption, which enhances the spectral response of the sensor to RH. Therefore, the sensitivity response is enhanced after the film coating is applied. Experimental results demonstrate that the proposed sensor achieves the maximum sensitivities to RH and temperature determined at −0.9261 nm/%RH and 0.0952 nm/°C, respectively. The sensor features a compact structure, high sensitivity and the ability to achieve dual-parameter sensing, which supports applications in biomedical, agricultural and electronic manufacturing fields.

## 1. Introduction

Relative humidity (RH) and temperature play a critical role in many industrial and scientific fields. For industries like biopharmaceuticals, food science, and electronics manufacturing, accurate measurement of these parameters is essential to maintain process control and product quality [1,2]. Conventional electrical sensors are very popular among the public because of their low price. However, they have significant drawbacks such as low sensitivity, electromagnetic interference susceptibility and large volume. In contrast, fiber optic sensors exhibit advantages including high sensitivity and compact structure [3,4]. These merits effectively alleviate the above limitations and make the sensors suitable for complex application scenarios.

Various fiber optic sensors have been developed for RH and temperature measurement. Most of the reported sensors are based on fiber Bragg gratings (FBGs) [5,6], Michelson interferometers (MIs) [7], Mach-Zehnder interferometers (MZIs) [8,9,10,11] and so on. Among these, MZIs are widely used in the measurement of RH and temperature because they are inexpensive and easy to fabricate [12]. As an example, Soltanian et al. demonstrated a variable waist-diameter tapered-fiber MZI aimed at dual-parameter RH and temperature detection [13]. The sensor achieved an RH response as high as 0.02 nm/%RH, along with a maximum temperature response of 0.5 nm/°C. Although this structure enables dual-parameter detection, its RH sensitivity remains relatively low. Cheng et al. demonstrated an inline microfiber interferometer using multi-core microfibers aimed at dual-parameter RH and temperature detection [14]. The sensor achieved an RH response of 0.06 nm/%RH, along with a temperature response of 0.004 nm/°C. Even though the RH sensitivity of the sensor has been enhanced, the sensor is costly because the multi-core fiber requires customization.

To address the problems in standard MZI structures, cascaded structures have been introduced. A cascaded sensing structure consisting of an FBG and a gap-coupled cladding waveguide MZI was fabricated by Pan et al. through femtosecond laser inscription [15]. This sensor was applied for RH and temperature measurement, with sensitivities of −0.08 nm/%RH and 0.01 nm/°C, respectively. In the same year, a structure consisting of an FBG embedded in a Sagnac interferometer (SI) was reported by Fan et al. [16]. This device exhibited an RH response of 0.09 nm/%RH and, from the FBG, a maximum temperature response of 0.01 nm/°C. Although these designs avoided the use of custom multi-core fiber, the cascaded FBG and MZI structure and the FBG embedded in the SI resulted in long sensor lengths, which were not suitable for practical applications.

Given the limitations mentioned above, researchers have modified sensors by coating humidity-sensitive materials to optimize sensing performance. As an example, a dual-parameter MZI sensor with a cobalt carbonate hydroxide hydrate nanosheet-array coating on the thin-core fiber region was developed by Tan et al. [17]. The RH response of the sensor is 0.56 nm/%RH and the temperature response is 1.29 nm/°C. While it achieved a significant sensitivity improvement over uncoated structures, the employment of special nanomaterial arrays led to high material costs, which limited the large-scale production and application. In another example, a core-offset MZI coated with polyvinyl alcohol (PVA) was developed by Dong et al. [18]. RH and temperature sensitivities of 0.26 nm/%RH and 0.15 nm/°C, respectively, were achieved with this sensor. Although PVA was employed to reduce material costs, the sensitivity of the sensor was sacrificed. Therefore, a coating structure based on a humidity-sensitive material is still required to achieve a balance between sensitivity and cost.

Demand has driven the exploration of various materials for humidity sensing. Natural polymers have emerged as promising candidates due to their biocompatibility and film-forming ability. Among them, chitosan has been demonstrated as an ideal natural-polymer humidity-sensitive material because of the favorable properties it possesses. It is a natural semicrystalline polysaccharide, and its porous structure contains amino (-NH_2_) and hydroxyl (-OH) groups [19]. The groups achieve reversible adsorption and desorption of water molecules via hydrogen bonds, so changes in ambient RH can alter the refractive index (RI) of chitosan [20]. Moreover, chitosan exhibits a range of structural and functional properties that render it highly suitable for the preparation of fiber-optic RH and temperature sensors. Chitosan possesses excellent film-forming ability and can form uniform thin films on diverse fiber structures through a dip-coating process [21]. Meanwhile, its features include non-toxicity, good biocompatibility, and high mechanical strength, all of which enable chitosan to satisfy the requirements of various sensing applications [22].

In this paper, a chitosan-coated MZI with a bamboo-shaped structure is proposed for RH and temperature measurement. The sensor is fabricated by using arc discharge to fuse between the no-core fiber and multimode fiber, which is then followed by taper treatment via an oxyhydrogen flame. The colloid is prepared from dissolved chitosan powder and employed as the humidity-sensitive material. The chitosan colloid is coated on the substrate structure to form a thin film, which improves the sensitivity of the sensor. Experimental results demonstrate that the sensor achieves a maximum RH sensitivity of −0.9261 nm/%RH. This sensor features high sensitivity, low crosstalk, and low cost, which supports its application in food processing, agricultural, and medical fields.

## 2. Fabrication Method and Working Principle

**Figure 1:**
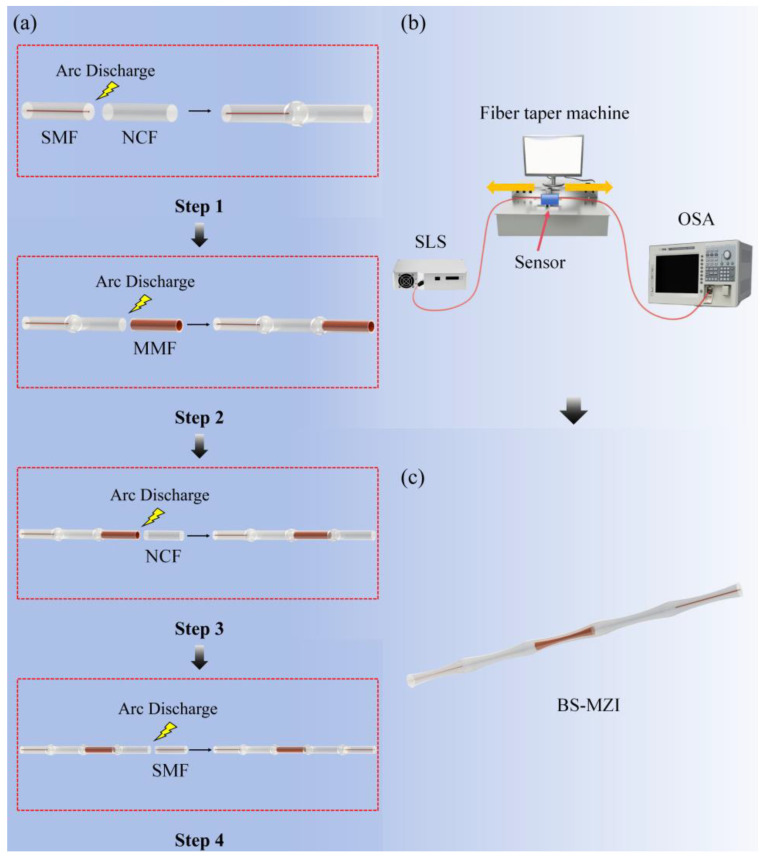
(**a**) The substrate structure fabrication steps. (**b**) The oxyhydrogen flame fiber-taper machine. (**c**) The schematic diagram of the BS-MZI.

Figure 1 shows the fabrication process and schematic diagram of the proposed bamboo-shaped MZI (BS-MZI). Specifically, Figure 1a illustrates the detailed fabrication steps for the substrate structure: in the first step, the prepared lead-in single mode fiber (SMF, 8.3/125 µm) was fused with the NCF (0/125 µm) via an arc fusion splicer (JiLong, KL-300T, Nanjing, China). After adjustment of the parameters (in Table 1) of the fusion splicer, multiple discharges were performed, and up-tapers were formed at the fusion joint. These tapers enhanced the mechanical strength of the structure and significantly improved the coupling efficiency. In the second step, the same fusion process and parameters as the first step were adopted. MMF (105/125 µm) segment was fused to the end of the NCF, and up-tapers were formed at the corresponding junction. In the third and fourth steps, the MMF and the lead-out SMF were successively connected to the end of the structure, finally forming an alternately spliced structure with up-tapers at each fusion joint. In the last step, the prepared structure was transferred to the oxyhydrogen flame fiber-taper machine (Shandong Coupler Technology Co., Ltd., Tai’an, China, AFBT-6000) for the tapering process, as shown in Figure 1b. The stretching speed was set to 150 μm/s, and the stretching time was 8 s. During the tapering process, light from a super-continuum light source (SLS, YSL Photonics Co., Ltd., SC-5, Wuhan, China) was launched into the sensor. Meanwhile, an optical spectrum analyzer (OSA, Yokogawa, Tokyo, Japan, AQ6370D) was connected to record the transmission spectra throughout fabrication. Finally, Figure 1c presents the schematic diagram of the proposed BS-MZI. To clarify the operating principle, the function of each fiber segment and the necessity of tapering are explained as follows. The first NCF lacks a light-guiding core, which allows the incident light to expand and excite multiple cladding modes. The subsequent MMF and the second NCF further modulate the mode distribution. The larger core diameter and higher numerical aperture of the MMF segment increase the number of excited cladding modes and enhance the coupling efficiency. Consequently, compared with a tapered structure without an MMF, the inclusion of the MMF yields stronger interference fringes and a higher extinction ratio (ER). The tapering process reduces the local fiber diameter and enhances the evanescent field [23], which strengthens the coupling between the core mode and the cladding modes. Without tapering, the mode coupling would be too weak to generate clear interference dips. Therefore, the proposed bamboo-shaped tapered structure not only improves the coupling efficiency but also produces distinct interference dips.

**Figure 2:**
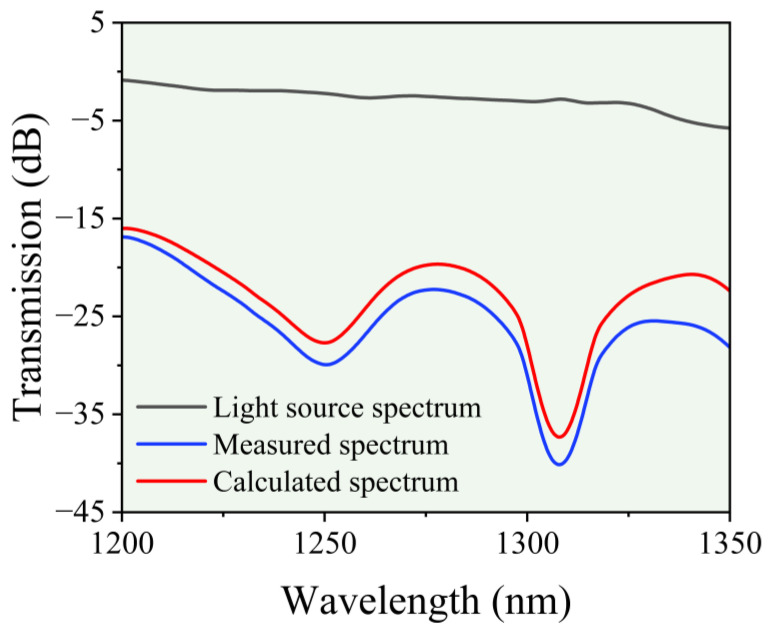
Optical spectra of SLS and the calculated spectrum after computational removal of source spectral fluctuations.

**Figure 3:**
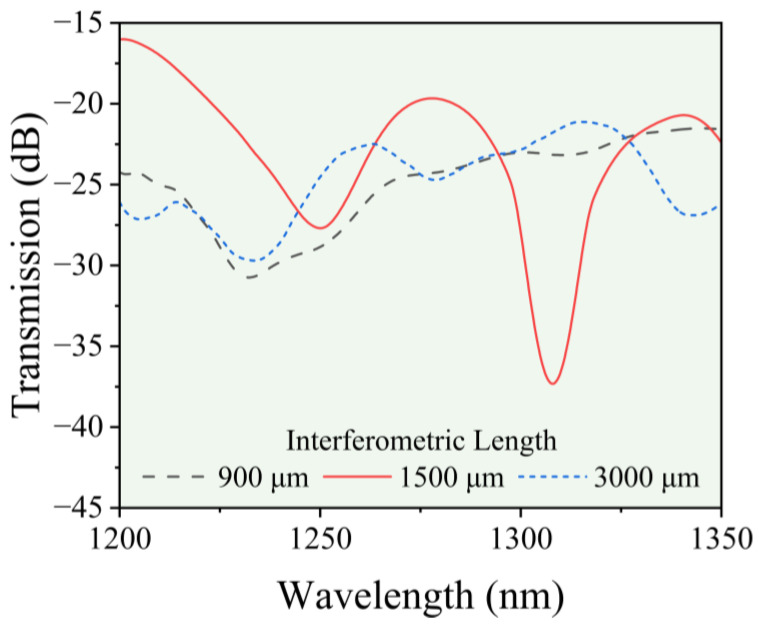
The spectra of the sensors with different interference lengths.

Figure 2 shows the optical spectra of the supercontinuum light source. Black curve represents the original output spectrum of the SLS. Blue curve is the spectrum recorded by the OSA after the light propagated through the sensor. Red curve is the difference between black curve and black curve, which is the effective spectrum used for the measurement in this study. This subtraction helps to eliminate the influence of the spectral bandwidth and the power distribution of the light source on the experimental results. For an MZI, the extinction ratio (ER) and free spectral range (FSR), as well as spectral visibility, are influenced by the interference length. Therefore, a quantitative investigation of the influence of the interference length is required to elucidate the relationship between the interference length and the MZI sensing performance. The transmission spectra for three samples with different interference lengths are shown in Figure 3. Specifically, the interference length refers to the length of the NCF-MMF-NCF segment of the BS-MZI. For a BS-MZI with an interference length of 900 μm, the ER and fringe visibility are limited. This limitation arises from insufficient mode coupling within the structure, and, therefore, the interference length needs to be increased. Further extension of the interference length of sensor to 3000 μm leads to excessive mode coupling, which degrades the spectral quality. Therefore, the interference length of the BS-MZI of 1500 μm is determined to be optimal, and this length was selected for the subsequent fabrication.

**Figure 4:**
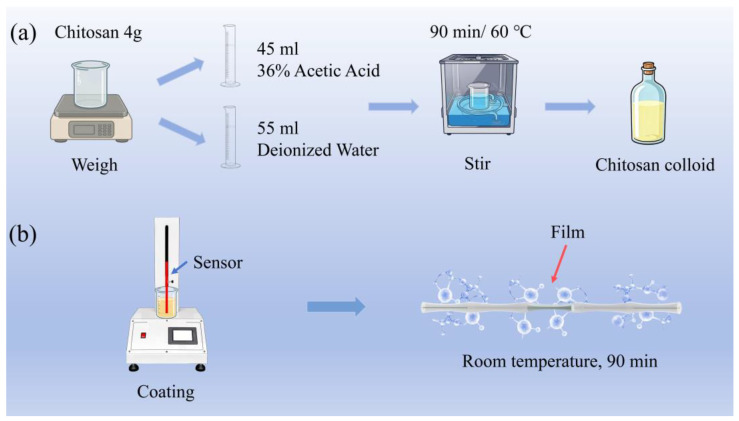
(**a**) Chitosan colloid preparation process. (**b**) The coating process of the chitosan colloid onto the structure.

The preparation and deposition of the coating material are critical for the BS-MZI. The preparation process of the chitosan colloid is shown in Figure 4a, and a 4% chitosan colloidal solution is used in this work. A total of 4 g of chitosan powder was dissolved in 45 mL of acetic acid solution, and then water was added to adjust the total mass to 100 mL. Sinopharm Chemical Reagent Co., Ltd. Shanghai, China supplied all chemicals used in this experiment. The solution was stirred in a water bath at 60 °C for 90 min to ensure thorough mixing. Subsequently, the solution was placed in a constant temperature incubator for 6 h, and the chitosan colloid was finally formed. The prepared chitosan colloid exhibits good film-forming ability and provides reliable support for the subsequent coating process.

**Figure 5:**
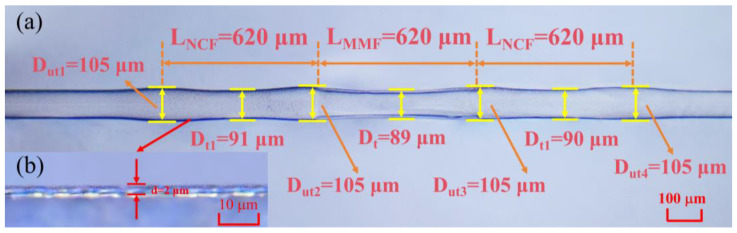
(**a**) Microscopic image of the BS-MZI structure. (**b**) Chitosan film coated onto the BS-MZI structure.

The coating process of the chitosan colloid is shown in Figure 4b. The sensor is immersed in the colloid and then withdrawn by a dip-coating machine. The film thickness is influenced by the withdrawal speed and the number of withdrawal cycles. A low withdrawal speed leads to a thin film with low water adsorption and a weak response. A high withdrawal speed or multiple withdrawal cycles results in an overly thick film, which degrades the spectral quality [24,25]. Through the above dip-coating process, the chitosan-film coating on the BS-MZI was successfully accomplished. Figure 5a presents a microscope image showing the fabricated structure. Based on the microscopic characterization, this sensor consists of four up-tapers and three tapers, which together form a bamboo-like shape. The unique shape maintains the mechanical strength and improves the coupling effect. The tapered structure is measured to have a length of 620 μm, an up-taper diameter of 105 μm, and a taper diameter of 90 μm. Specifically, the taper diameter is the average of three individual taper measurements, with an error of 1 μm. The diameters of the individual tapers in the sensor are relatively consistent, which demonstrates good fabrication consistency. A microscope image of the chitosan-film layer is shown in Figure 5b, and the film is relatively uniform, with a thickness of 2 μm. The uniform thickness provides a foundation for the stability of subsequent RH measurements.

**Figure 6:**
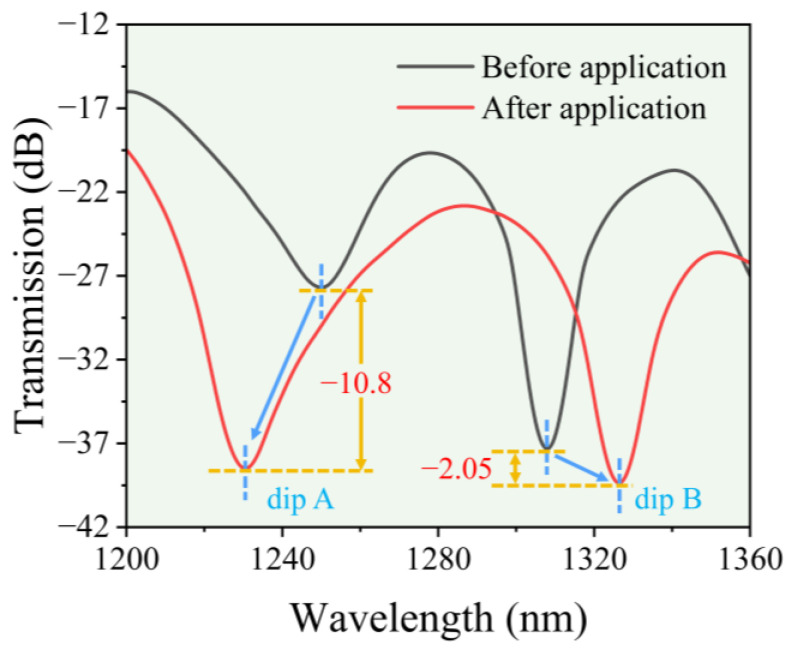
The transmission spectra of the BS-MZI before and after chitosan coating.

Figure 6 presents a comparison spectra of the BS-MZI before and after the chitosan-coating application. After chitosan coating, the ER of dip A increased from 27.7 dB to 38.5 dB. This represents an increase of 10.8 dB, a relative improvement of 38.99%, and a 12.02-fold reduction in the minimum transmission. For dip B, the ER increased from 37.30 dB to 39.35 dB, which is an increase of 2.05 dB, a relative improvement of 5.5%, and a 1.60-fold reduction in the minimum transmission. Compared with the uncoated BS-MZI, the coated structure exhibited more appropriate ER and FSR, and the spectral resolution was also remarkably improved. This result established a favorable spectral foundation for the subsequent RH and temperature measurements.

**Figure 7:**
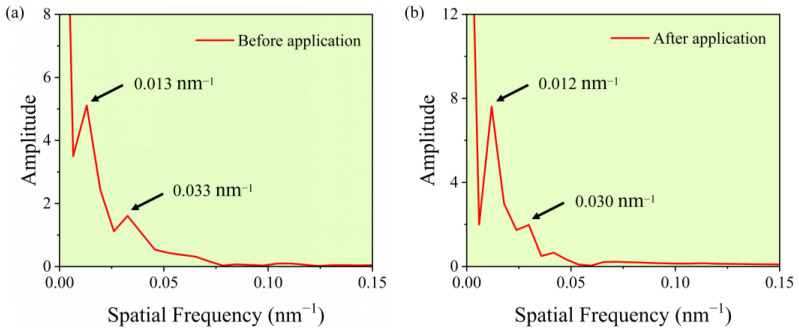
(**a**) FFT spectrum of the structure before coating. (**b**) FFT spectrum of the structure after coating.

To enable a more accurate identification of the spectral components before and after the coating process, Figure 7 shows the FFT-processed spectra of the BS-MZI obtained from its original optical spectra. Through the FFT spectra, the periodic characteristics and frequency constituents contained in the optical spectra can be analyzed. After the film dip-coating, the low-frequency components in the FFT spectrum became more prominent. This result indicates that the film, applied by dip-coating on the BS-MZI, significantly modified its effective refractive index (ERI). The regular shift of the main interference components in the FFT spectrum indicates that the interference mechanism is not disrupted. This means that the coating does not introduce scattering loss or coherence changes caused by surface irregularities. It shows that the primary role of the coating is to modulate the effective refractive index of the modes rather than to introduce strong random scattering.

**Figure 8:**
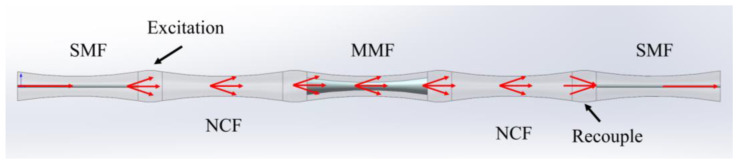
The light propagation path within the BS-MZI.

A schematic diagram of the light propagation path within the BS-MZI is illustrated in Figure 8. The tapering process reduces the fiber cladding diameter, and, thus, the confinement of light by the fiber core is weakened. Higher-order modes are excited at the up-taper between the lead-in SMF and the NCF, and further excitation occurs at each subsequent taper and up-taper. The final taper allows these modes to pass through, after which they re-couple at the last up-taper, and then enter the core of the lead-out SMF.

Although the proposed BS-MZI contains multimode and no-core fiber sections with multiple taper and up-taper perturbations, these structures mainly serve as mode converters and mode filters. Therefore, the final interference spectrum can be interpreted as an equivalent two-mode interference process, which is dominated by the fundamental core mode and one principal higher-order cladding mode. Therefore, the working principle of the BS-MZI can be simplified to the two-beam interference, which can be theoretically explained as follows [26]:

(1) $$I=I_{1}+I_{2}+2\sqrt{I_{1}I_{2}}\cos\varphi$$

where *I*_1_ and *I*_2_ denote the intensities of the two interfering paths in the MZI, and *φ* denotes the phase difference between the two interferometric arms. This phase difference originates from the interference between the fundamental core mode and the higher-order cladding modes in the MMF, and it can be formulated as follows [27]:

(2) $$\varphi =\frac{2\pi \Delta nL}{\lambda }$$

where Δ*n* is the ERI difference between the two interferometric arms, *L* is the total interaction length, and *λ* is the incident light wavelength. The phase-matching condition is given by

(3) $$\varphi =\left(2m+1\right)\pi ,m=1,2,3,\ldots$$

Under this condition, the resonant wavelengths of *m*-th order mode (*λ_m_*) corresponding to the interference dips in the BS-MZI can be expressed as [28]

(4) $$\lambda_{m}=\frac{2}{2m+1}\Delta n\cdot L$$

After the structure was coated with chitosan, the ERI of the outer cladding region was modified. When the environmental humidity varies, the -NH_2_ and -OH groups within the chitosan layer capture water molecules via hydrogen bonding [20]. This process induces chitosan swelling and lowers the RI of the coating, which, in turn, reduces Δ*n*. According to Equation (4), the reduction in Δ*n* results in a blue shift of the resonant dip wavelength. This shift converts the humidity-induced RI change into a measurable spectral response. Thus, it is realized that external humidity variations are converted into spectral shifts. Moreover, when the ambient temperature varies within the non-denaturation range of the material, the RI is mainly modulated by the thermo-optic effect of the structure. When the temperature increases, Δ*n* is raised through this effect, and Equation (4) shows that an increase in λ*_m_* causes the resonant wavelength of the sensor to red shift. This response mechanism establishes a basis for simultaneous temperature and RH measurement.

**Figure 9:**
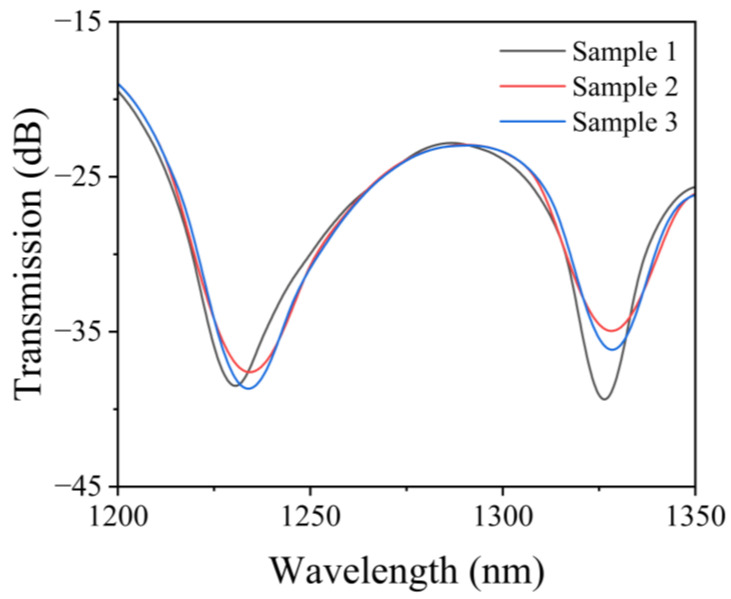
Spectra of three samples fabricated with optimized parameters.

Given the critical importance of fabrication repeatability for fiber-optic sensors, it is necessary to perform experimental verification by comparing the spectra of multiple devices. As shown in Figure 9, the spectra of three samples, fabricated and coated under identical parameters and conditions, exhibit nearly unchanged resonant peak wavelengths. This result demonstrates excellent fabrication stability of the sensor.

## 3. Experimental Results and Sensing Analysis

**Figure 10:**
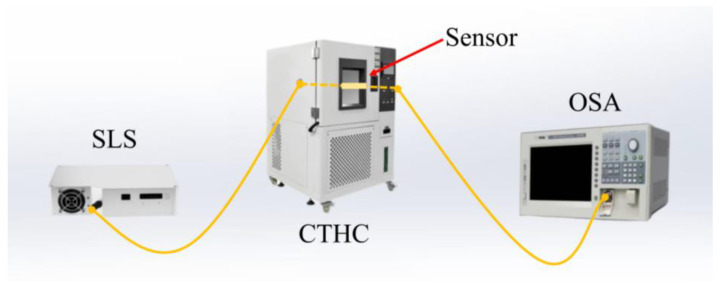
Schematic diagram of the experimental configuration for sensor performance measurement.

To evaluate the humidity-sensing performance of the proposed sensor, experimental verification of the RH response was carried out. The experimental setup for measurement is shown in Figure 10. First, the sensor was placed in a constant temperature and humidity chamber (CTHC) and tested at room temperature with 5% RH intervals. The OSA was used to record the spectral wavelength shifts of the structure.

**Figure 11:**
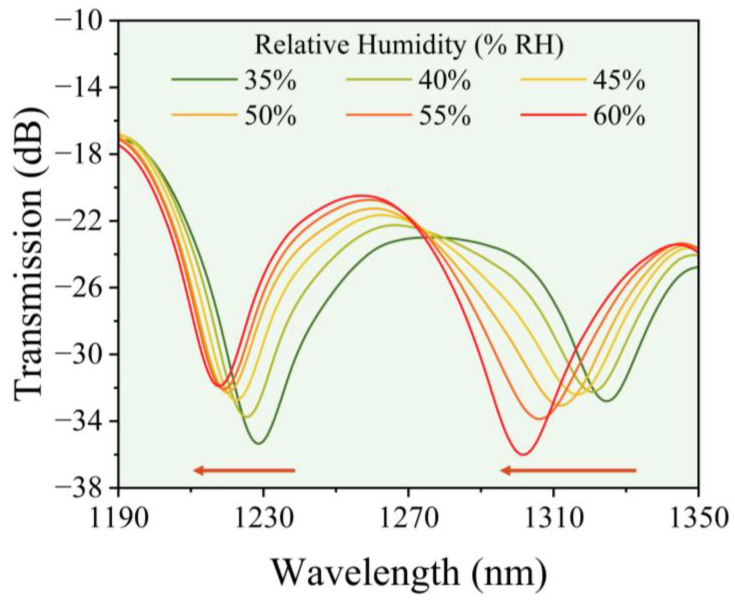
Transmission spectra of the coated MZI structure as a function of RH.

**Figure 12:**
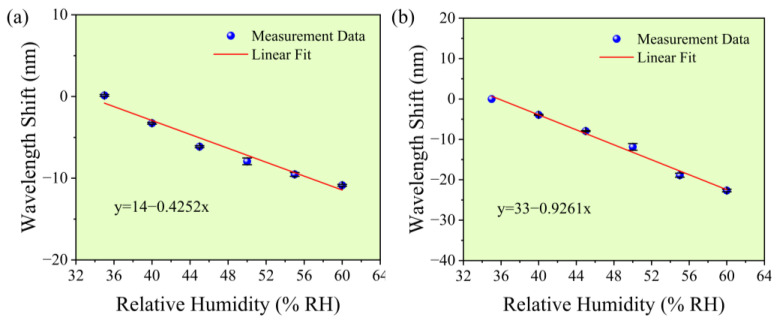
(**a**) RH sensitivity of dip A in the BS-MZI. (**b**) RH sensitivity of dip B in the BS-MZI.

Figure 11 displays the sensor spectra obtained under various humidity levels. Both dip A and dip B of the sensor exhibited a blue shift in the wavelength as the RH increased from 35% to 60%. This phenomenon aligns with the theoretical expectation of the hygroscopic swelling effect. Such swelling induces changes in the ERI of the sensing region and results in a measurable shift in the interference spectrum. The testing range of 35–60% RH was selected primarily according to the normal humidity operational requirements of the sensor in common application scenarios, including biopharmaceutical storage, food processing, and so on. This RH range covers the typical interval for long-term stable monitoring in the above scenarios. At low RH levels, the water uptake of the chitosan film is limited, the swelling effect is small, and, consequently, the sensitivity is reduced. Under near-saturation RH conditions, the film is expected to approach its maximum water absorption capacity and the wavelength response becomes nonlinear and eventually saturates. Such a linear spectral response within the tested range reflects the stable humidity-sensing capability of the proposed structure. It also verifies the rationality of the designed BS-MZI sensing structure. Figure 12 shows the linear fitting of the RH-wavelength response for the two resonant dips based on three independent measurements conducted at two-day intervals, with error bars representing the repeatability and long-term stability. The small error bars in the measurement mainly originate from minor environmental disturbances, such as airflow-induced mechanical vibrations of the sensing structure. However, the magnitude of these errors is very small. Therefore, they can be considered to have no detectable influence on the measurement accuracy. Consequently, the proposed sensor remains capable of reliable dual-parameter measurement. In the range of 35% to 60% RH, the two resonant dips in the sensor showed RH sensitivities of −0.4252 nm/%RH and −0.9261 nm/%RH, respectively. These linear fits correspond to the expressions y = 20 − 0.4252x and y = 46 − 0.9261x, where x is the relative humidity (%RH) and y is the absolute wavelength (nm). The intercept (20 nm and 46 nm) represents the theoretical wavelength at 0% RH, that is, the total blue shift extrapolated to dry conditions. The slope (−0.4252 nm/%RH and −0.9261 nm/%RH) denotes the humidity sensitivity, meaning that, for every 1% RH increase, the wavelength shifts toward shorter wavelengths by the corresponding amount. The obtained sensitivities demonstrate the capability of the sensor for high-sensitivity RH measurement.

**Figure 13:**
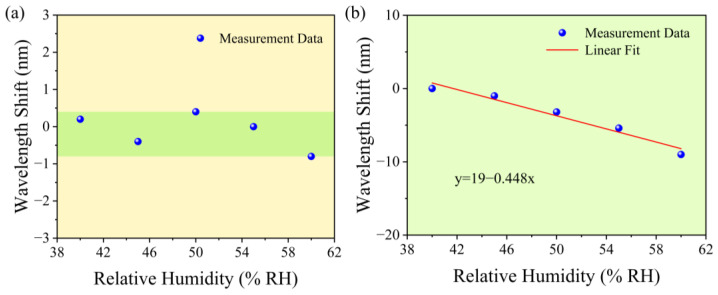
(**a**) RH sensitivity of the sensor with a 0.8 μm-thick film. (**b**) RH sensitivity of the sensor with a 3.2 μm-thick film.

Considering that different film thicknesses have distinct effects on the sensing performance of the BS-MZI, it is necessary to investigate the sensor performance under various film thicknesses. The two additional sensors, with chitosan-film thicknesses of 0.8 μm and 3.2 μm, were fabricated under the same substrate parameters. The RH responses of the two sensors within the measurement range of 40% to 60% are presented in Figure 13. It can be observed that no significant wavelength shift of the resonant dip occurs for the sensor with a chitosan-film thickness of 0.8 µm, while for the sensor with a film thickness of 3.2 µm, its RH sensitivity reaches −0.448 nm/%RH. An appropriate film thickness yields optimal performance, and, therefore, a thickness of 2 µm is considered suitable. This result is consistent with the theoretical analysis presented earlier.

**Figure 14:**
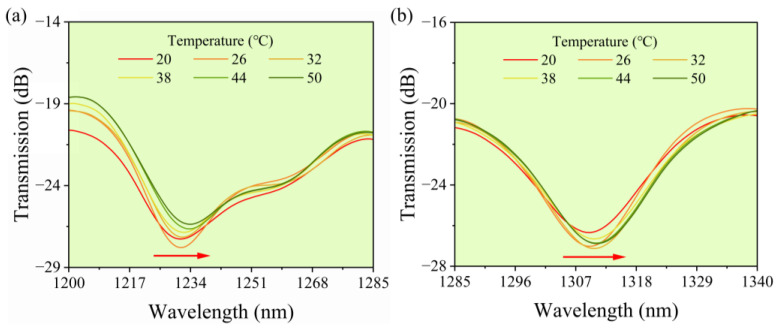
(**a**) Transmission spectra of the BS-MZI structure for dip A under different temperatures. (**b**) Transmission spectra of the BS-MZI structure for dip B under different temperatures.

**Figure 15:**
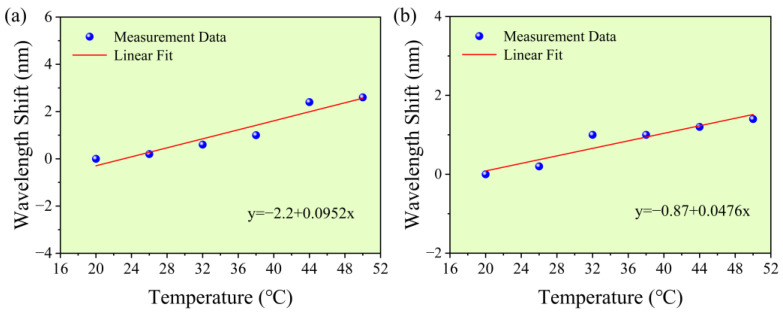
(**a**) Temperature sensitivity of dip A in the BS-MZI. (**b**) Temperature sensitivity of dip B in the BS-MZI.

However, in practical applications, ambient temperature fluctuations can interfere with RH measurements in environmental monitoring and other scenarios. Therefore, to further ensure the measurement accuracy of the sensor in complex environments, temperature characterization is also required. At a constant room RH, the proposed sensor was tested for its temperature response. Figure 14 presents the transmission spectra of the sensor recorded at different temperatures. When the temperature was adjusted from 20 °C to 50 °C at 6 °C intervals, the sensor shows a red shift in wavelength for both dip A and dip B. The results present a linear relationship between wavelength shift of the BS-MZI and temperature variation. Linear fitting yields temperature sensitivities of 0.0952 nm/°C for dip A and 0.0476 nm/°C for dip B, as shown in Figure 15. This linear temperature response provides support for matrix demodulation in dual-parameter sensing.

For the proposed dual-parameter sensor, it is essential to separate the contributions of temperature and RH to the spectrum. Among various methods, the matrix demodulation approach is well-suited for MZI structures. The MZI provides the advantage of possessing multiple resonance dips. This feature allows demodulation of the contributions of both parameters without the introduction of additional temperature or RH compensation structures. This matrix can be established based on the two given dips in the BS-MZI [29]:

(5) $$\left(\begin{array}{c} \Delta \lambda_{A}\\ \Delta \lambda_{B} \end{array}\right)=\left(\begin{array}{cc} S_{RH,A} & S_{T,A}\\ S_{RH,B} & S_{T,B} \end{array}\right)\left(\begin{array}{c} \Delta RH\\ \Delta T \end{array}\right)$$

where Δ*λ_A_* and Δ*λ_B_* are the wavelength shifts of the dips. *S_RH,A_* and *S_RH,B_* represent the sensitivity coefficients of dip *A* and dip *B* to *RH* variations, respectively. Similarly, *S_T,A_* and *S_T,B_* denote the sensitivity coefficients for dip *A* and dip *B* with respect to temperature variations, in that order. Δ*T* and Δ*RH* stand for the changes in temperature and *RH*, which can be determined through matrix inversion

(6) $$\left(\begin{array}{c} \Delta RH\\ \Delta T \end{array}\right)={\left(\begin{array}{cc} -0.4274 & 0.0952\\ -0.9314 & 0.0476 \end{array}\right)}^{-1}\left(\begin{array}{c} \Delta \lambda_{A}\\ \Delta \lambda_{B} \end{array}\right)$$

Based on the matrix operation presented in Equation (6), the cross-influence between RH and temperature can be effectively suppressed, and synchronous measurement of the two parameters is, thereby, achieved.

To compare the performance of the proposed BS-MZI, a comparative analysis with relevant reported sensors is performed. Table 2 summarizes the temperature sensitivity, *RH* sensitivity, the involved sensitive materials, and minimum detectable resolution of the reported *RH* and temperature sensors. It is clear that most of sensors suffer from limitations in terms of sensitivity. Although the cobalt carbonate hydroxide hydrate-based fiber optic sensor with an MZI configuration achieves high sensitivity, the humidity-sensitive coating is limited by high material costs and complex fabrication. In contrast, the proposed chitosan-coated BS-MZI sensor exhibits competitive relative-humidity sensitivity, relatively low temperature crosstalk, low humidity-measurement resolution and low cost. These characteristics are favorable for practical applications such as electronics manufacturing.

## 4. Conclusions

A dual-parameter fiber sensor coated with chitosan and based on a bamboo-shaped interferometric structure is proposed. The BS-MZI is fabricated through arc discharge, a tapering process, and a dip-coating process. The optimal interference length and coating thickness of the sensor are determined experimentally. The temperature and RH responses of the BS-MZI are measured, and the results show that the sensor exhibits a maximum temperature and RH sensitivities of 0.0952 nm/°C and −0.9261 nm/%RH, respectively. The temperature and RH responses of the BS-MZI are decoupled via the matrix method, and this approach enables simultaneous and accurate measurement of the two parameters. With its dual-parameter sensing capability, high sensitivity, and low cost, the proposed sensor is promising for applications in environmental monitoring, biomedicine, and other related fields.

## Figures and Tables

**Table 1:** The parameters of the fusion splicer.

Parameter	Value
Discharge Power	130 bit
Discharge Time	2200 ms
Discharge Overlap Length	30 μm

**Table 2:** Comparison of different sensors with simultaneous measurement of RH and temperature.

Sensor Type	RH Sensitivity (pm/%RH)	Temperature Sensitivity (pm/°C)	Coating Material	Minimum Detectable Resolution (%RH)	Ref.
FBG	18	−80	None	0.056	[5]
MI	3.8	14.3	Polymeric microsphere	5.26	[7]
MZI	25.6	−109.9	Ultraviolet glue	0.78	[10]
MZI	59.8	4.2	None	0.33	[14]
MZI-FBG	−97.3	11.04	None	0.21	[15]
MZI	560	1290	Cobalt carbonate hydroxide hydrate	0.036	[17]
MZI	256	153	PVA	0.078	[18]
MZI	−926.1	95.2	Chitosan	0.022	This work

## Data Availability

The data presented in this study are available on request from the corresponding author.
